# The effects of course length on freestyle swimming speed in elite female and male swimmers – a comparison of swimmers at national and international level

**DOI:** 10.1186/2193-1801-2-643

**Published:** 2013-12-01

**Authors:** Mathias Wolfrum, Beat Knechtle, Christoph Alexander Rüst, Thomas Rosemann, Romuald Lepers

**Affiliations:** Institute of General Practice and for Health Services Research, University of Zurich, Zurich, Switzerland; Cardiovascular Center Cardiology, University Hospital Zürich, Zürich, Switzerland; Gesundheitszentrum St. Gallen, St. Gallen, Switzerland; INSERM U1093, Faculty of Sport Sciences, University of Burgundy, Dijon, France; Facharzt FMH für Allgemeinmedizin, Gesundheitszentrum St. Gallen, Vadianstrasse 26, 9001 St. Gallen, Switzerland

**Keywords:** Athlete, Sex, Endurance, Performance

## Abstract

Freestyle swimming performance over 50 m, 100 m, 200 m, 400 m, 800 m and 1,500 m was compared on short (25 m) and long (50 m) course for 92,196 national swimmers (*i.e.* annual high score list Switzerland) and 1,104 international swimmers (*i.e.* finalists FINA World Championships) from 2000 to 2012. National and international swimmers of both sexes were on average 2.0 ± 0.6% faster on short than on long course. Sex-related differences in swimming speed were greater on short than on long course for international and national swimmers from 50 m to 800 m. Freestyle swimming performance improved across years for international swimmers in both short- and long-course whereas only male national swimmers were able to improve on short and long course events except for short course events on 800 m and 1,500 m. Performance in national women competing in short and long course events showed only improvements on 50 m, 100 m and 1,500 m across years. The sex-related differences in freestyle swimming performance showed no change for international swimmers. For national swimmers, the sex-related differences in freestyle swimming performance increased over time in long course from 50 m to 800 m, but decreased for 1,500 m. In conclusion, elite female and male freestyle swimmers at national and international level were about 2% faster on 25 m compared to 50 m course. During the 2000–2012 period, international as well as national swimmers (*i.e.* for national level predominantly men) improved freestyle swimming performance in both long and short course. More vigorous and optimized training programs focused on muscular force production in combination with efficient swimming skills might close the performance gap between elite swimmers at national level and FINA finalists. Further research especially including effects of anthropometric, biomechanical, and physiological factors is required to fully understand the effects of course length on freestyle swimming performance, and to determine whether course length has similar effects on other swim styles.

## Background

National and international swimming competitions are held on either short (25 m) or long (50 m) course (2012a). Swimming speeds are generally faster for short-course events (FINA, 2012b), due to the greater number of turns made for any given swimming distance (Keskinen et al. 1996; Telford et al. 1988; Wakayosh et al. 1999). Turns provide increased propulsion and moderate exercise recuperation, resulting in several physiological and biomechanical differences between short- and long-course events, such as a lower heart rate and lower blood lactate levels in freestyle swimming events on short courses (Keskinen et al. 2007; Lowenstein et al. 1994; Telford et al. 1988).

Effects of course length on swimming performance differ to some extent between men and women (Wirtz et al. 1992). Male freestyle swimmers gain more advantage from short-course events than their female counterparts, because men are able to reach higher speeds during turns (Wirtz et al. 1992). However, the sex-related differences in freestyle swimming performance have only been compared for short and long courses in 50 m events; most of the previous studies investigating sex-related differences in swimming speed only used results of long-course events.

Despite predictions that performance in freestyle swimming had reached human limits (Nevill et al. 2007) new records were set on long courses during the 2012 Olympic Games, and on short courses during the 2012 FINA World Championships (FINA, 2012b), suggesting that freestyle swimming performance is still improving. However, few studies have investigated recent temporal trends in freestyle swimming (Johnson et al. 2009; Seiler et al. 2007; Telford et al. 1988) and no previous study analysed temporal trends in freestyle swimming for all official race distances on both short and long courses.

The present study used annual results of national (*i.e.* Swiss) swimmers and bi-annual results of international (*i.e.* finalists in FINA World Championships) swimmers. Since women do not compete in FINA World Championships of 1,500 m distance on short courses, we compared data of national and international swimmers. The aims of the study were to investigate (*i*) the effects of course length on freestyle swimming speed for both men and women at national and international level and (*ii*) the changes in freestyle swimming speed during the 2000–2012 period. We hypothesized that (*i*) swimming speed would be faster on short course than on long course for both sexes and (*ii*) swimming speeds would increase over time for both short- and long-course at both national and international level.

## Methods

### Samples and sources

All procedures used in the study met the ethical standards of the Swiss Academy of Medical Sciences (http://www.samw.ch/en/Ethics/Guidelines/Currently-valid-guidelines.html) and were approved by the Institutional Review Board of Kanton St. Gallen, Switzerland with a waiver of the requirement for informed consent of the participants given the fact that the study involved the analysis of publicly available data.

Annual top ten results of freestyle swimmers at national level for all freestyle events on 25 m and 50 m courses were analyzed for men and women from the Swiss high score list and bi-annual results of freestyle finalists in the FINA World Swimming Championships for swimmers at international level during the 2000–2012 period. The data for national swimmers were obtained from the Swiss Swimming Federation (http://rankings.fsn.ch/) and for international swimmers from the Fédération Internationale de Natation (FINA) (http://www.fina.org).

Short-course race results at national level were available for 45,888 Swiss swimmers (*i.e.* 22,216 women and 23,672 men), and at international level for 527 FINA World Championship finalists (*i.e.* 239 women and 288 men). Long-course race results at national level were available for 46,308 Swiss swimmers (*i.e.* 22,257 women and 24,051 men), and at international level for 577 FINA World Championship finalists (*i.e.* 289 women and 288 men).

### Data processing

All race times were converted to swimming speed in order to compare results at different distances (*i.e.* 50 m, 100 m, 200 m, 400 m, 800 m, and 1,500 m) using the equation [swimming speed in m/s] = [race distance in m]/[race time in s]. Swimming speed of the annual ten fastest female and male Swiss swimmers and of the eight women and eight men competing in the finals of the FINA World Championships for each race distance and year were used to compare performance on short and long course. For this analysis, swimming speeds were pooled over time, providing a sample size of 120 for Swiss and 96 for FINA swimmers, for each course length, sex, and distance. Due to the low number of athletes competing in 1,500 m freestyle races, analyses for this distance used only the five fastest swimming speeds by each sex (*n* = 60 for each data point).

Sex-related differences in swimming speed were calculated as the absolute value of ([woman’s swimming speed] – [man’s swimming speed])/[man’s swimming speed] × 100, for pairs of equally placed athletes (*e.g.*, 1^st^ place woman and 1^st^ place man, 2^nd^ place woman and 2^nd^ place man, etc*.*). The mean and standard deviation were then computed for the pairs.

### Statistical analyses

Each data set was tested for normal distribution and homogeneity of variances prior to further statistical analysis. Normal distribution was tested using a D’Agostino and Pearson omnibus normality test. Homogeneity of variances was tested using Levene’s test in analyses with two groups and with Bartlett’s test in analyses with more than two groups. To find significant changes in swimming speed across years, linear regression analysis was used (Model 1). A hierarchical regression model was implemented to avoid the influence of a cluster-effect (*i.e.* when athletes finished more than once) on the results for the annual top or annual top three (Model 2). In detail an individual and bijective identification number (ID) was assigned to every athlete, thus the individual ID appeared multiple times if an athlete finished more than once within the annual top or annual top three and could be used as a maker for ‘groups’ (*i.e.* cluster) of identical athletes. Regression analyses of performance were further corrected for age of athletes to prevent a misinterpretation of ‘age-effect’ with ‘time-effect’ by adding the age of an athlete as a correction factor to Model 2 (Model 3). Student’s *t*-test was used to determine the significance of differences between the performance in long and short course. One-way analysis of variance (ANOVA) with subsequent Dunnett post-hoc analysis was used to determine the significance of differences between more than two groups. A two-way-ANOVA was used to examine the interactive effect of sex and course length on performance. Statistical analyses were performed using IBM SPSS Statistics (Version 19, IBM SPSS, Chicago, IL, USA) and GraphPad Prism (Version 5, GraphPad Software, La Jolla, CA, USA). Significance was accepted at *p* < 0.05 (two-tailed for *t*-tests). Results are reported as mean ± standard deviation in the text and figures.

## Results

### Effects of course length on swimming speed

**Figure 1:**
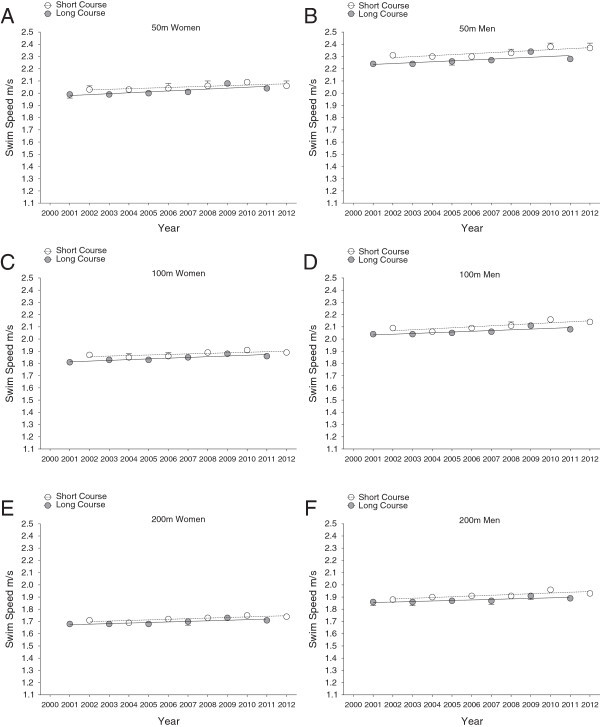
**Changes in swimming speed of female and male FINA World Champion finalists at international level during each year from 2000 to 2012 in short course (25 m)** ***versus*** **long course (50 m) for 50m in women (Panel A) and men (Panel B), for 100m in women (Panel C) and men (Panel D) and for 200m in women (Panel E) and men (Panel F) (Mean ± SD).** The figure is based on linear regression model 1.

**Figure 2:**
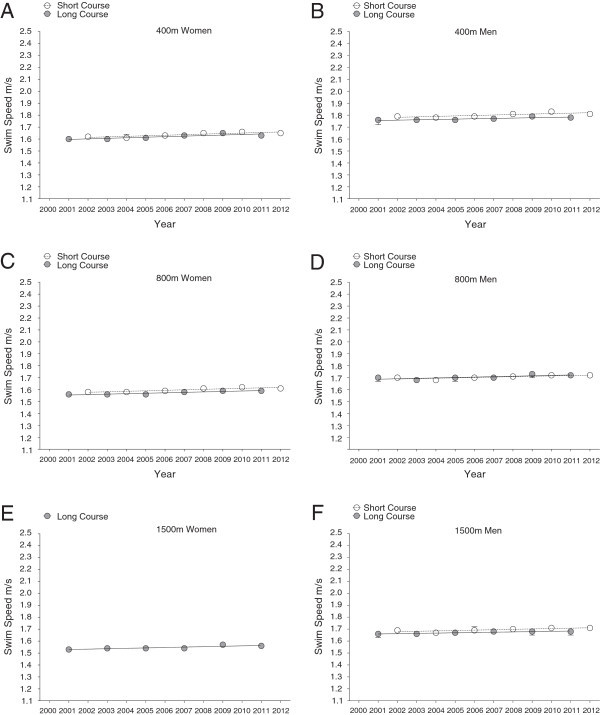
**Changes in swimming speed of female and male FINA World Champion finalists at international level during each year from 2000 to 2012 in short course (25 m)** ***versus*** **long course (50 m) for 400m in women (Panel A) and men (Panel B), for 800m in women (Panel C) and men (Panel D) and for 1,500m in women (Panel E) and men (Panel F) (Mean ± SD).** *Note*: women do not compete in 1,500 m short course at FINA World Championships. The figure is based on linear regression model 1.

**Figure 3:**
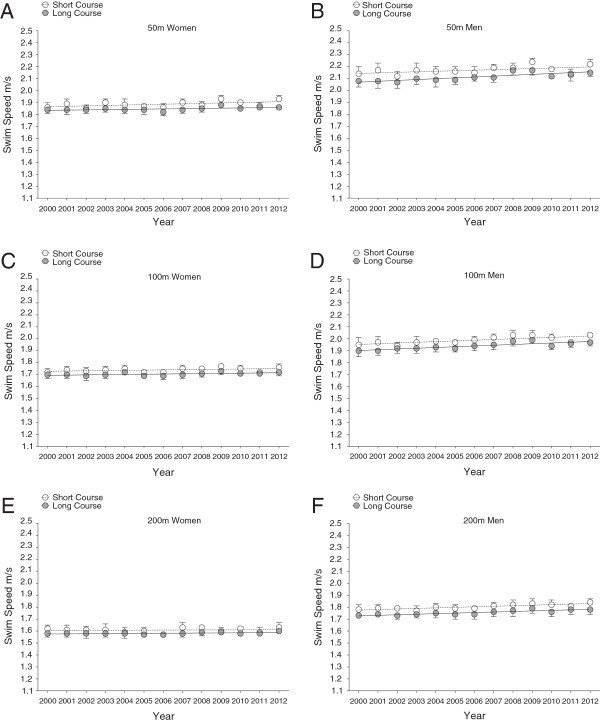
**Changes in swimming speed of top ten Swiss women and men at national level during each year from 2000 to 2012 in short course (25 m)** ***versus*** **long course (50 m) for 50m in women (Panel A) and men (Panel B), for 100m in women (Panel C) and men (Panel D) and for 200m in women (Panel E) and men (Panel F) (Mean ± SD).** The figure is based on linear regression model 1.

**Figure 4:**
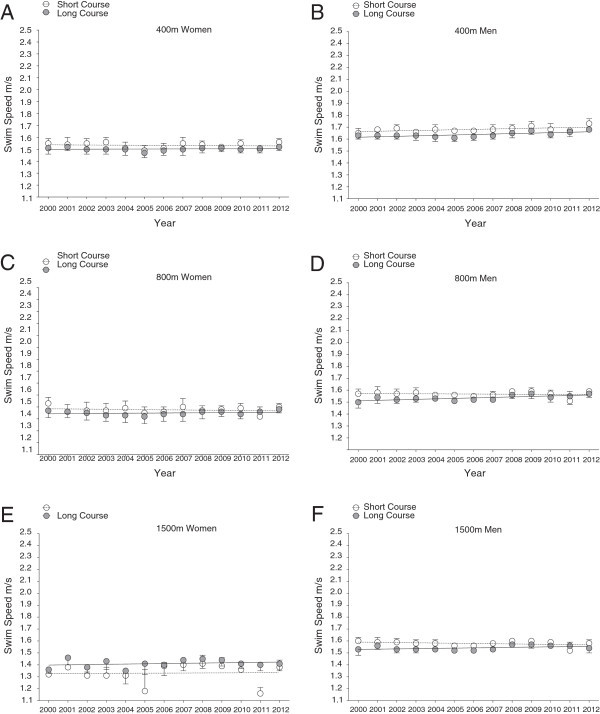
**Changes in swimming speed of top ten Swiss women and men at national level during each year from 2000 to 2012 in short course (25 m)** ***versus*** **long course (50 m) for 400m in women (Panel A) and men (Panel B), for 800m in women (Panel C) and men (Panel D) and for 1,500m in women (Panel E) and men (Panel F) (Mean ± SD).** The figure is based on linear regression model 1.

Freestyle swimming speeds of national and international men and women were faster on short course than on long course (Table 1). There were two exceptions where national women were significantly faster on long than on short course in 1,500 m (long course 1.54 ± 0.01 m · s^–1^*versus* short course 1.45 ± 0.01 m · s^–1^, *P* < 0.01), and international men had equal swimming speeds in 800 m races on both long and short course. Men were in general faster than women at international and national competition level (Figures 1, 2, 3 and 4). These findings were also highlighted by a two-way analysis of variance, which demonstrated a positive main effect of male sex and short course event on swim speed (Table 2). Furthermore, sex and course length had positive interactive effects on swimming speed in national swimmers competing in 50 m, 200 m, 800 m, and 1,500 m, and on swimming speed in international swimmers competing in 50 m, 400 m, and 800 m; with a positive interactive-effect of male sex and short course on swimming speed. There is no data for race distance of 1,500 m at international competition level, as women do not compete in FINA World Championships at this particular race distance on short courses. Swimming speed consistently decreased with increasing race distance, and men were faster than women (Figures 1, 2, 3 and 4).

**Table 1:** **Absolute and percent differences in swimming speed (short course 25 m - long course 50 m) for men and women competing at national and international level over different distances**

	Absolute difference (m · s^-1^)	Percent difference (%)	***p*** ^*^
**Men at national level**			
50 m	0.05 ± 0.00	2.10 ± 0.19	< 0.0001
100 m	0.05 ± 0.01	2.64 ± 0.39	< 0.0001
200 m	0.03 ± 0.02	1.79 ± 0.99	0.048
400 m	0.04 ± 0.02	2.39 ± 0.94	0.01
800 m	0.03 ± 0.01	2.11 ± 0.30	0.0005
1500 m	0.03 ± 0.01	2.12 ± 0.30	< 0.0001
**Men at international level**			
50 m	0.06 ± 0.01	2.59 ± 0.25	< 0.0001
100 m	0.05 ± 0.01	2.50 ± 0.38	< 0.0001
200 m	0.04 ± 0.01	1.84 ± 0.49	< 0.0001
400 m	0.03 ± 0.01	1.58 ± 0.40	< 0.0001
800 m	-0.01 ± 0.01	0.63 ± 0.63	0.13
1500 m	0.03 ± 0.01	1.45 ± 0.31	< 0.0001
**Women at national level**			
50 m	0.06 ± 0.01	2.84 ± 0.40	< 0.0001
100 m	0.04 ± 0.01	2.35 ± 0.36	< 0.0001
200 m	0.04 ± 0.00	2.65 ± 0.17	< 0.0001
400 m	0.03 ± 0.00	2.10 ± 0.25	< 0.0001
800 m	0.03 ± 0.01	2.10 ± 0.40	0.0003
1500 m	-0.09 ± 0.02	-6.43 ± 1.55	< 0.0001
**Women at international level**			
50 m	0.03 ± 0.01	1.47 ± 0.51	0.0035
100 m	0.03 ± 0.01	1.73 ± 0.36	0.0005
200 m	0.03 ± 0.01	1.53 ± 0.53	< 0.0001
400 m	0.02 ± 0.00	1.14 ± 0.19	0.0003
800 m	0.02 ± 0.01	1.35 ± 0.38	0.0003

Mean ± standard deviation. *p–value for absolute difference between short and long course.

**Table 2:** **Statistical significance (2-way ANOVA) of effects of sex, course length, swim distance, the interactive effects of sex × course length on swimming speed of national and international freestyle swimmers**

	Results of two-way ANOVA (sex × course length)		
	Sex	Course length	Sex × course length
**National level**			
50 m	*F* = 4840.0, *p* < 0.0001	*F* = 129.6, *p* < 0.0001	*F* = 14.4, *p* = 0.0002
100 m	*F* = 4883.4, *p* < 0.0001	*F* = 164.8, *p* < 0.0001	*F* = 2.0, *p* = 0.16
200 m	*F* = 3110.4, p < 0.0001	*F* = 153.6, p < 0.0001	*F* = 9.6, p = 0.002
400 m	*F* = 1770.5, p < 0.0001	*F* = 103.2, p < 0.0001	*F* = 2.1, p = 0.15
800 m	*F* = 418.1, p < 0.0001	*F* = 46.5, p < 0.0001	*F* = 8.4, p = 0.024
1500 m	*F* = 1178.9, p < 0.0001	*F* = 6.3, p = 0.012	*F* = 56.8, p < 0.0001
**International level**			
50 m	*F* = 3192.9, *p* < 0.0001	*F* = 98.9, *p* < 0.0001	*F* = 7.6, *p* = 0.007
100 m	*F* = 186.9, *p* < 0.0001	*F* = 5.91, *p* < 0.016	*F* = 0.1, *p* = 0.76
200 m	*F* = 3233.9, p < 0.0001	*F* = 95.7, p < 0.0001	*F* = 3.4, p = 0.068
400 m	*F* = 3683.5, p < 0.0001	*F* = 89.1, p < 0.0001	*F* = 8.4, p = 0.004
800 m	*F* = 2667.1, p < 0.0001	*F* = 29.2, p < 0.0001	*F* = 29.2, p < 0.0001

### Temporal changes in swimming speed and sex-related differences on short and long courses

Men and women finalists in FINA World Championships showed significant improvements in swimming speed from 2000 to 2012 in both short and long course and over all distances (Figures 1 and 2, Tables 3 and 4). Male national swimmers were also able to improve performance during this period on short and long course events, except for short course events on longer distances (800 and 1500 m; Figures 3 and 4, Tables 5 and 6). Performance in national women competing in short and long course events showed only improvements on short race distances (50 and 100 m) and the very long distances (1,500 m) across years. These temporal trends of swimming speed remained unchanged when corrected for multiple participations (Model 2, Tables 3, 4, 5 and 6) and age of athletes with multiple participations (Model 3).

**Table 3:** **Multi-level regression analyses for change in swimming speed for 50 m, 100 m, and 200 m in swimmers at international level across years for women and men (Model 1) with correction for multiple participations (Model 2) and age of athletes with multiple participations (Model 3)**

Model	***ß***	SE ( ***ß*** )	Stand. ***ß***	T	***p***
**50 m women long course**					
1	0.007	0.001	0.635	5.568	< 0.001
2	0.007	0.001	0.635	5.568	< 0.001
3	0.007	0.001	0.620	5.510	< 0.001
**50 m women short course**					
1	0.006	0.001	0.511	3.983	< 0.001
2	0.006	0.001	0.511	3.983	< 0.001
3	0.006	0.001	0.510	3.930	< 0.001
**50 m men long course**					
1	0.007	0.001	0.634	5.557	< 0.001
2	0.007	0.001	0.634	5.557	< 0.001
3	0.007	0.001	0.630	5.466	< 0.001
**50 m men short course**					
1	0.008	0.001	0.645	5.721	< 0.001
2	0.008	0.001	0.645	5.721	< 0.001
3	0.008	0.001	0.660	5.647	< 0.001
**100 m women long course**					
1	0.006	0.001	0.768	8.126	< 0.001
2	0.006	0.001	0.768	8.126	< 0.001
3	0.006	0.001	0.769	8.060	< 0.001
**100 m women short course**					
1	0.004	0.001	0.527	4.200	< 0.001
2	0.004	0.001	0.527	4.200	< 0.001
3	0.004	0.001	0.541	4.240	< 0.001
**100 m men long course**					
1	0.006	0.001	0.701	6.674	< 0.001
2	0.006	0.001	0.701	6.674	< 0.001
3	0.006	0.001	0.697	6.575	< 0.001
**100 m men short course**					
1	0.007	0.001	0.668	6.094	< 0.001
2	0.007	0.001	0.668	6.094	< 0.001
3	0.007	0.001	0.667	5.947	< 0.001
**200 m women long course**					
1	0.005	0.001	0.680	6.292	< 0.001
2	0.005	0.001	0.680	6.292	< 0.001
3	0.005	0.001	0.683	6.233	< 0.001
**200 m women short course**					
1	0.005	0.001	0.571	4.714	< 0.001
2	0.005	0.001	0.571	4.714	< 0.001
3	0.005	0.001	0.577	4.759	< 0.001
**200 m men long course**					
1	0.005	0.001	0.519	4.120	< 0.001
2	0.005	0.001	0.519	4.120	< 0.001
3	0.005	0.001	0.521	4.091	< 0.001
**200 m men short course**					
1	0.006	0.001	0.651	5.823	< 0.001
2	0.006	0.001	0.651	5.823	< 0.001
3	0.006	0.001	0.650	5.738	< 0.001

*ß* = regression coefficient, Stand*. ß* = standardized coefficient.

**Table 4:** **Multi-level regression analyses for change in swimming speed for 400 m, 800 m, and 1,500 m in swimmers at international level across years for women and men (Model 1) with correction for multiple participations (Model 2) and age of athletes with multiple participations (Model 3)**

Model	***ß***	SE ( ***ß*** )	Stand. ***ß***	T	***p***
**400 m women long course**					
1	0.005	0.001	0.701	6.663	< 0.001
2	0.005	0.001	0.701	6.663	< 0.001
3	0.005	0.001	0.711	6.678	< 0.001
**400 m women short course**					
1	0.004	0.001	0.604	5.134	< 0.001
2	0.004	0.001	0.604	5.134	< 0.001
3	0.004	0.001	0.620	5.052	< 0.001
**400 m men long course**					
1	0.003	0.001	0.395	2.913	0.006
2	0.003	0.001	0.395	2.913	0.006
3	0.003	0.001	0.418	3.107	0.003
**400 m men short course**					
1	0.004	0.001	0.566	4.660	< 0.001
2	0.004	0.001	0.566	4.660	< 0.001
3	0.004	0.001	0.566	4.629	< 0.001
**800 m women long course**					
1	0.004	0.001	0.540	4.395	< 0.001
2	0.004	0.001	0.540	4.395	< 0.001
3	0.004	0.001	0.547	4.299	< 0.001
**800 m women short course**					
1	0.004	0.001	0.635	5.580	< 0.001
2	0.004	0.001	0.635	5.580	< 0.001
3	0.004	0.001	0.599	5.127	< 0.001
**800 m men long course**					
1	0.004	0.001	0.448	3.398	0.001
2	0.004	0.001	0.448	3.398	0.001
3	0.003	0.001	0.423	2.973	0.005
**800 m men short course**					
1	0.004	0.001	0.590	4.960	< 0.001
2	0.004	0.001	0.590	4.960	< 0.001
3	0.004	0.001	0.584	4.893	< 0.001
**1,500 m women long course**					
1	0.003	0.001	0.482	3.730	0.001
2	0.003	0.001	0.482	3.730	0.001
3	0.003	0.001	0.448	3.224	0.002
**1,500 m men long course**					
1	0.002	0.001	0.326	2.342	0.024
2	0.002	0.001	0.326	2.342	0.024
3	0.002	0.001	0.344	2.403	0.020
**1,500 m men short course**					
1	0.003	0.001	0.498	3.895	< 0.001
2	0.003	0.001	0.498	3.895	< 0.001
3	0.003	0.001	0.492	3.822	< 0.001

*ß* = regression coefficient, Stand*. ß* = standardized coefficient.

**Table 5:** **Multi-level regression analyses for change in swimming speed for 50 m, 100 m, and 200 m in swimmers at national level across years for women and men (Model 1) with correction for multiple participations (Model 2) and age of athletes with multiple participations (Model 3)**

Model	***ß***	SE ( ***ß*** )	Stand. ***ß***	T	***p***
**50 m women long course**					
1	0.002	0.001	0.218	2.524	0.013
2	0.002	0.001	0.218	2.524	0.013
3	0.001	0.001	0.179	2.086	0.039
**50 m women short course**					
1	0.003	0.001	0.334	4.011	< 0.001
2	0.003	0.001	0.334	4.011	< 0.001
3	0.003	0.001	0.266	3.381	< 0.001
**50 m men long course**					
1	0.007	0.001	0.491	6.368	< 0.001
2	0.007	0.001	0.491	6.368	< 0.001
3	0.007	0.001	0.488	6.647	< 0.001
**50 m men short course**					
1	0.005	0.001	0.330	3.956	< 0.001
2	0.005	0.001	0.330	3.956	< 0.001
3	0.005	0.001	0.363	4.612	< 0.001
**100 m women long course**					
1	0.002	0.001	0.237	2.765	0.007
2	0.002	0.001	0.237	2.765	0.007
3	0.002	0.001	0.220	2.613	0.010
**100 m women short course**					
1	0.002	0.001	0.279	3.285	0.001
2	0.002	0.001	0.279	3.285	0.001
3	0.002	0.001	0.268	3.233	0.002
**100 m men long course**					
1	0.006	0.001	0.536	7.182	< 0.001
2	0.006	0.001	0.536	7.182	< 0.001
3	0.006	0.001	0.528	7.234	< 0.001
**100 m men short course**					
1	0.006	0.001	0.495	6.445	< 0.001
2	0.006	0.001	0.495	6.445	< 0.001
3	0.006	0.001	0.472	6.392	< 0.001
**200 m women long course**					
1	0.002	0.001	0.205	2.371	0.019
2	0.002	0.001	0.205	2.371	0.019
3	0.002	0.001	0.220	2.647	0.009
**200 m women short course**					
1	0.001	0.001	0.056	0.640	0.524
2	0.001	0.001	0.056	0.640	0.524
3	0.001	0.001	0.119	1.357	0.177
**200 m men long course**					
1	0.004	0.001	0.427	5.337	< 0.001
2	0.004	0.001	0.427	5.337	< 0.001
3	0.005	0.001	0.440	5.462	< 0.001
**200 m men short course**					
1	0.005	0.001	0.451	5.715	< 0.001
2	0.005	0.001	0.451	5.715	< 0.001
3	0.005	0.001	0.465	5.918	< 0.001

*ß* = regression coefficient, Stand*. ß* = standardized coefficient.

**Table 6:** **Multi-level regression analyses for change in swimming speed for 400 m, 800 m, and 1,500 m in swimmers at national level across years for women and men (Model 1) with correction for multiple participations (Model 2) and age of athletes with multiple participations (Model 3)**

Model	***ß***	SE ( ***ß*** )	Stand. ***ß***	T	***p***
**400 m women long course**					
1	0.001	0.001	0.076	0.863	0.390
2	0.001	0.001	0.076	0.863	0.390
3	0.001	0.001	0.090	1.128	0.261
**400 m women short course**					
1	-.001	0.001	-0.064	-0.726	0.469
2	-.001	0.001	-0.064	-0.726	0.469
3	-8.258E-005	0.001	-0.007	-0.085	0.932
**400 m men long course**					
1	0.004	0.001	0.418	5.212	< 0.001
2	0.004	0.001	0.418	5.212	< 0.001
3	0.004	0.001	0.414	5.467	< 0.001
**400 m men short course**					
1	0.003	0.001	0.305	3.625	< 0.001
2	0.003	0.001	0.305	3.625	< 0.001
3	0.003	0.001	0.344	4.055	< 0.001
**800 m women long course**					
1	0.001	0.001	0.090	1.018	0.311
2	0.001	0.001	0.090	1.018	0.311
3	0.001	0.001	0.101	1.262	0.209
**800 m women short course**					
1	-0.001	0.001	-0.092	-1.050	0.296
2	-0.001	0.001	-0.092	-1.050	0.296
3	-0.001	0.001	-0.058	-0.685	0.495
**800 m men long course**					
1	0.004	0.001	0.404	4.992	< 0.001
2	0.004	0.001	0.404	4.992	< 0.001
3	0.004	0.001	0.397	5.026	< 0.001
**800 m men short course**					
1	-0.001	0.001	-0.084	-0.957	0.340
2	-0.001	0.001	-0.084	-0.957	0.340
3	-0.001	0.001	-0.075	-0.879	0.381
**1,500 m women long course**					
1	0.006	0.002	0.255	2.971	0.004
2	0.006	0.002	0.255	2.971	0.004
3	0.006	0.002	0.256	3.224	0.002
**1,500 m women short course**					
1	0.005	0.002	0.195	2.099	0.038
2	0.005	0.002	0.195	2.099	0.038
3	0.005	0.002	0.197	2.100	0.038
**1,500 m men long course**					
1	0.003	0.001	0.314	3.742	< 0.001
2	0.003	0.001	0.314	3.742	< 0.001
3	0.003	0.001	0.314	3.853	< 0.001
**1,500 m men short course**					
1	0.000	0.001	-0.017	-0.197	0.844
2	0.000	0.001	-0.017	-0.197	0.844
3	0.000	0.001	-0.019	-0.229	0.819

*ß* = regression coefficient, *Stand. ß* = standardized coefficient.

Sex-related differences in swimming speed of national swimmers competing in races from 50 m to 800 m on long course increased significantly during 2000 to 2012, but decreased in 1,500 m. Sex-related differences in swimming speed of international swimmers showed no change over time in any events, regardless of distance or course length.

## Discussion

The main findings of the present study were, firstly, that elite national and international freestyle swimmers were on average 2.0 ± 0.6% faster on short course than on long course. Exceptions were national women with faster swimming speeds in 1,500 m long course events than in short course and international men with faster swimming speeds in 800 m long course than in short course. Secondly, swimming speed of international men and women increased significantly in short and long course during the 2000–2012 period, whereas only men national swimmers were able to improve on short and long course events. Thirdly, sex-related differences in swimming speed increased over time for national swimmers except for 1,500 m distance but did not change significantly for international swimmers at any course length and distance.

### Faster swimming speed in freestyle in short course

The results of the present study generally supported the hypothesis that swimming speeds of male and female freestyle swimmers would be faster on short course than on long course. These results confirm results of previous studies examining effects of course length on performance in 200 m freestyle (Keskinen et al. 2007; Lowenstein et al. 1994). In those races, swimmers spend about twice as long turning and gliding in 25 m pools than in 50 m pools (*i.e.* 31.5 s *versus* 13.5 s, on average) (Keskinen et al. 2007). Each turn results in recovery time leading to a decrease in lactate production and an increase in lactate clearance in the upper body and arm muscles which are used for a regular stroke (Craig, 1986; Keskinen et al. 2007; Lowenstein et al. 1994; Telford et al. 1988; Wirtz et al. 1992). Swimmers with a good turning performance have a particularly large advantage in short course. Men with higher peak leg power than women gain more advantage from turning and benefit more from swimming on short course (Dore et al. 2005). Thus, percent differences in swimming speeds tended to be greater for international men than for international women (Table 1). However, the trend was not clear in swimmers at national level.

### Decrease in sex-related differences with increasing race distance in freestyle in short and long course

The decrease in the sex-related difference in swimming speed observed with increasing distance is well known for freestyle events on long course, and is attributed to an increasing economy of female swimmers with increasing race distance (Tanaka and Seals 1997). Greater economy in women is due to smaller body size, less body drag, greater percent fat, lower body density, and shorter lower limbs, resulting in a more horizontal and streamlined swim position compared to male swimmers (Hinrichs 2007; Lavoie and Montpetit 1986; Pendergast et al. 1977; Toussaint et al. 1988). Greater swimming economy in women could provide an additional advantage in long course where phases of regular stroke are twice as long as in short course (Keskinen et al. 2007). This might explain our finding that national female swimmers were even faster on long course than on short course in 1,500 m freestyle.

### Different temporal trends at international and national level across years

The hypothesis that freestyle swimming performance would improve over time was supported by the results for FINA finalists in both long and short course and for national men over long course, which was not surprising in light of freestyle swimming records set in recent events (FINA, 2012b). Stanula et al. (2012) identified an even longer trend showing that performance in Olympic freestyle swimming events of 50 m to 1,500 m on long course improved during 1896 to 2008.

Improved swimming performance is partly attributable to technological advances, such as deeper, deck-level pools, more effective anti-wave lane ropes, and improved swim suits (FINA, 2010; Nevill et al. 2007). The lack of fundamental improvement in national swimmers, compared to that in international swimmers could reflect less use of advanced technologies or less intense training (Mujika, 1998; Mujika et al. 1995). Particularly short course performance is more dependent on start and turning times than stroking, and therefore, requires more complex technical skills than long course performance (Keskinen et al. 2007; Smith et al. 2002). Both less use of advanced technologies and less intense swim training might also have contributed to the consistently slower performance of Swiss swimmers. FINA regulated the use of full-body, polyurethane swimsuits in 2009, and the Swiss Swimming Federation followed this regulation. Interestingly, no Swiss national record in freestyle swimming on short course has been broken since 2009 for men and 2008 for women (Schweizer Schwimmverband, 2012).

In addition to technological advances, improved swimming techniques and/or training methods, and increased access to the sport by a larger number of athletes probably contributed to the observed improvement in swim performance (Schulz and Curnow 1988; USA-Swimming, 2011). Psychology and motivation, which also affect athletic performance, might differ between national and international level competitors explaining differences of temporal trends in swimming performance (Johnson et al. 2009; Miller 1993).

Male and female FINA finalists showed similar improvement in swimming speed during 2000–2012, which explains the lack of a change in sex-related differences. Nevill et al. (2007) similarly reported that the sex-related differences in various swimming and running events were remarkably stable during the last 60 years. However, our results further showed that the sex-related difference in Swiss long-course events increased over time, because the performance of Swiss men improved, while performance of Swiss women did not. The sex-related difference in Swiss short-course events did not change, because neither men nor women showed improvement on short course. Less improvement over time in Swiss than in FINA swimmers might be a result of the fewer athletes competing at the national level, reducing competitive pressure and selection for faster swimmers.

### Limitation, implications for future research and practical applications

Interpretation of these results is limited to some extent by the observational and cross-sectional study design. Moreover possible influences of anthropometric (Kukolj et al. 1999; Latt et al. 2010; Zampagni et al. 2008), biomechanical (Keskinen et al. 2007; Latt et al. 2010), and physiological (Latt et al. 2010) factors were not considered. However, this drawback is compensated for by the large study population, which provided sufficient power to detect small differences between groups that were statistically significant even after implementation of various statistical models to correct for potential confounders, such as multiple participations of athletes and age. Additional studies are required to determine whether course length has similar effects on other swim styles. These studies should include anthropometric, biomechanical, and physiological factors to fully understand the effects of course length on swim performance. For the first time our data provide extensive evidence over the complete range of official freestyle race distances that pool length plays a tremendous role in determining freestyle swimming performance. The results of the present study further indicate that performance in freestyle swimming is still improving at international level while remained unchanged national. Swimmers at national level should aim at more vigorous and optimized training programs to close the performance gap between elite national and international performance level.

## Conclusion

Freestyle swimmers at both national and international level were on average 2.0 ± 0.6% faster on short course than on long course. Faster freestyle swimming speeds on 25 m course than on 50 m course has been widely acknowledged, but not previously demonstrated using extensive results for elite male and female swimmers over the full range of official race distances. International freestyle swimmers showed a consistent improvement in freestyle swimming performance during the 2000–2012 period, whereas only male national swimmers were able to improve on both short and long course events. Performance in national women competing in short and long course events showed only improvements on 50 m, 100 m and 1,500 m across years. More vigorous and optimized training programs, focused on muscular force production in combination with efficient swimming skills, might close the performance gap between elite Swiss swimmers and FINA finalists. Further research, especially including effects of anthropometric, biomechanical, and physiological factors, is required to fully understand the effects of course length on freestyle swimming performance, and to determine whether course length has similar effects on other swim styles.
